# Supplementary material for: Novel tools for comparing the architecture of psychopathology between neurogenetic disorders: An application to X- vs. Y-chromosome aneuploidy effects in males
Source: medRxiv. 2025 Jan 13:2025.01.10.25320352. Preprint. [Version 2] doi: 10.1101/2025.01.10.25320352 (PMC12191080; doi:10.1101/2025.01.10.25320352)
Supplement: Supplement 1 [file media-1.pdf]

SUPPLEMENT

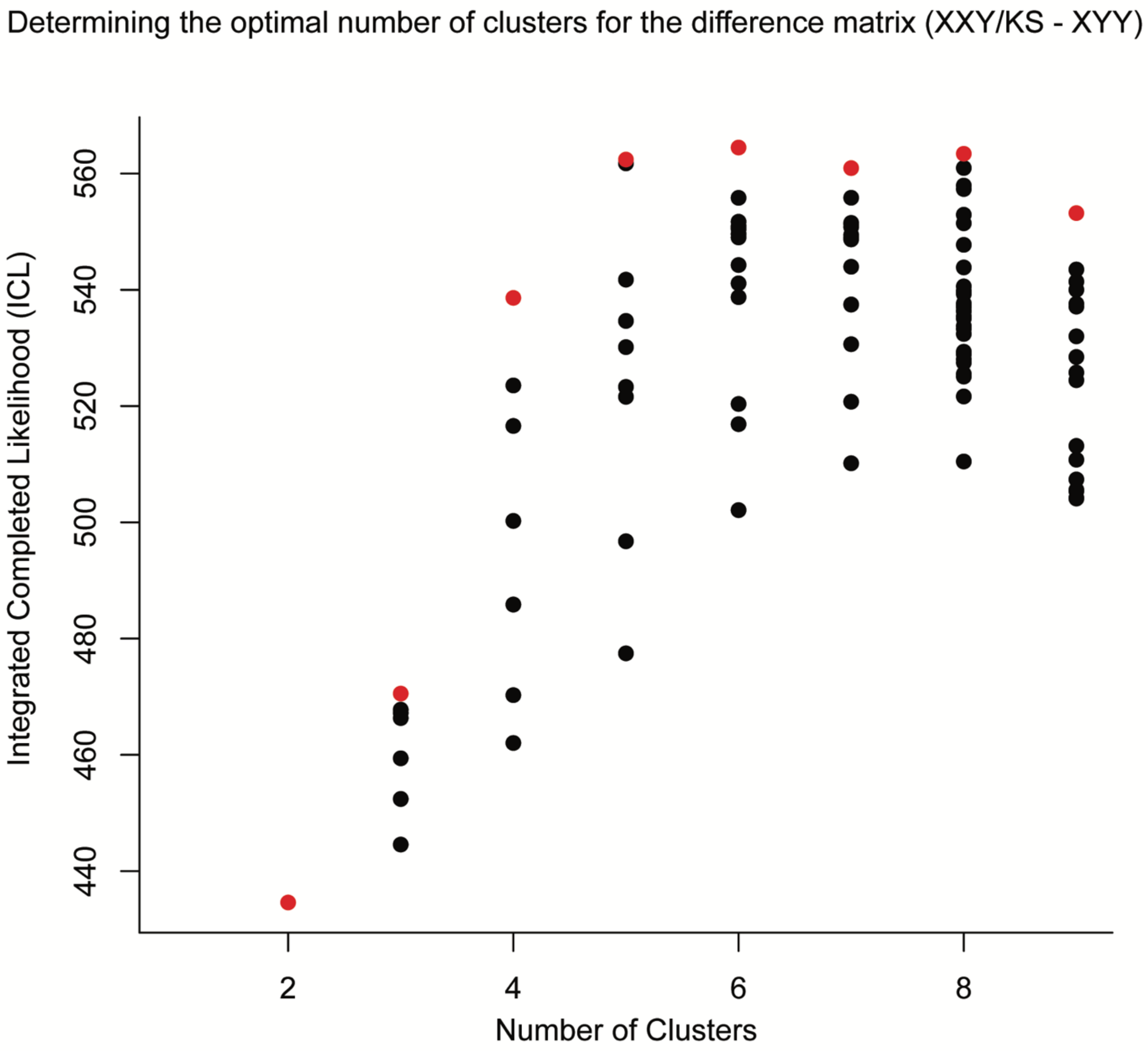

Figure S1: Optimal cluster determination. Plot depicting the integrated completed likelihood (ICL) value across iterations of the Weighted Stochastic Block Model. The optimal number of clusters was defined as the number that maximized the ICL value (clusters = 6 for the difference matrix).

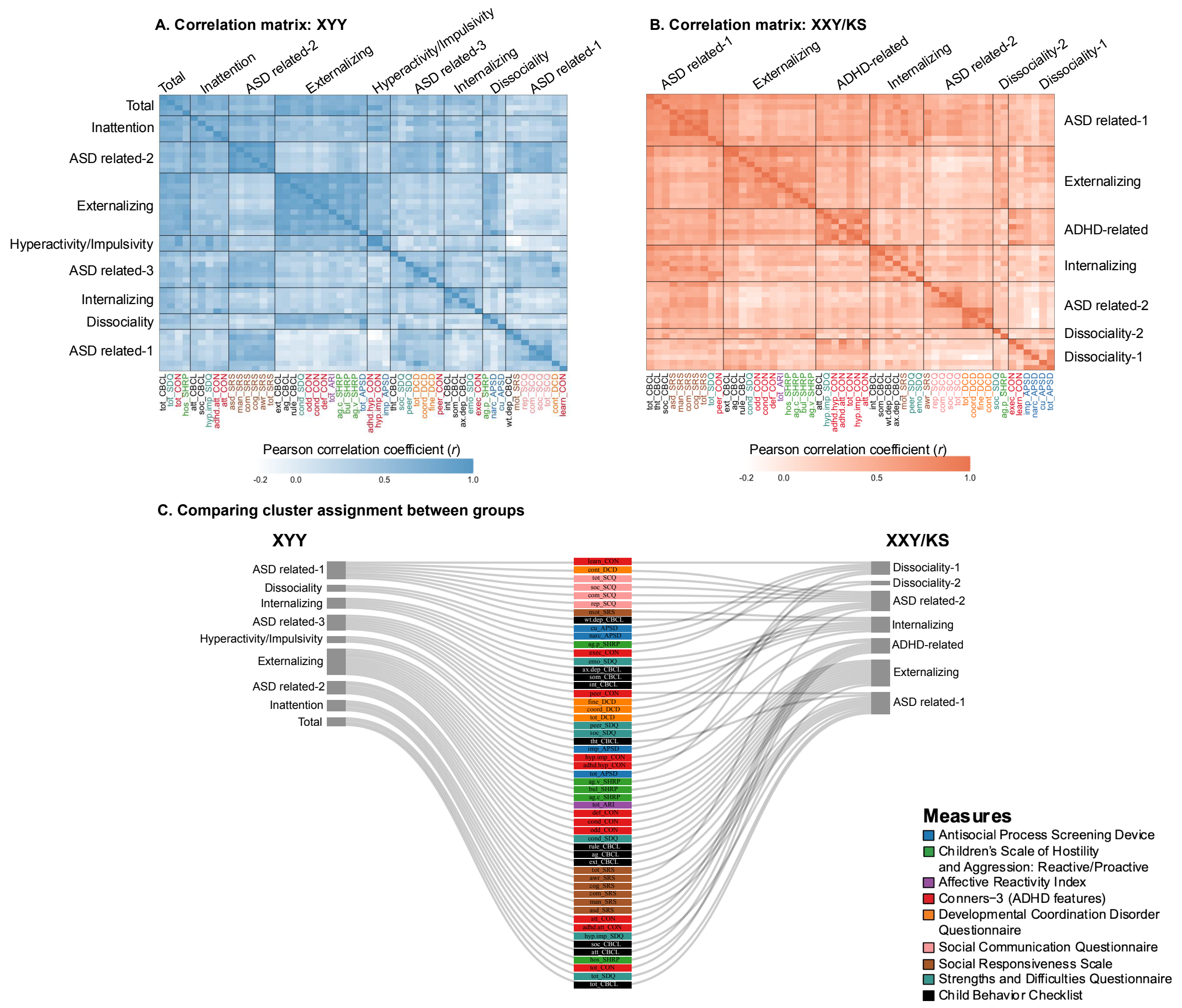

Figure S2: Fine-grained symptom correlation and domain-level coupling for XXY/KS and XYY syndrome. (A) Heatmap depicting the weighted stochastic block modeling (WSBM) solution of nine clusters (outlined in black) for the Pearson Correlation Coefficients between scales for XYY syndrome. **(B)** Heatmap depicting the weighted stochastic block modeling (WSBM) solution of seven clusters (outlined in black) for the Pearson Correlation Coefficients between scales for XXY/KS. The hue intensity depicts the magnitude of group differences in correlation between each unique pair of scales. **(C)** Sankey diagram depicting the cluster membership of the 53 scales in XYY syndrome versus XXY/KS. Scale names are color-coded to the instrument (see **Table S1**).

Table S1

Intrument (Abbreviation)	Subscale	Variable Name	*n* (XXY/KS, XY)	*n* (XYY, XY)	Mean age (XXY/KS, XY)	Mean age (XYY, XY)	Global coupling (XXY/KS)	Global coupling (XYY)	Deming residual	Divergence score	*p*-value against null distribution	Absolute delta effect size (XXY/KS - XYY)
**Antisocial Process Screening Device (APSD)**	Total	tot_APSD	101, 73	58, 55	16.21, 17.17	13.24, 13.78	0.35	0.48	0.11	0.23	0.208	0.72
	Callous/Unemotional	cu_APSD	101, 73	58, 55	16.21, 17.17	13.24, 13.78	0.29	0.28	-0.03	0.54	0.453	1.07
	Narcissism	narc_APSD	101, 73	58, 55	16.21, 17.17	13.24, 13.78	0.17	0.36	0.18	0.25	0.308	0.58
	Impulsivity	imp_APSD	101, 73	58, 55	16.21, 17.17	13.24, 13.78	0.30	0.40	0.07	0.18	0.628	0.25
**Children's Scale of Hostility and Aggression (SHRP)**	Verbal aggression	ag.v_SHRP	100, 38	54, 45	16.16, 12.46	12.13, 11.46	0.42	0.54	0.09	0.16	0.474	1.28
	Bullying	bul_SHRP	100, 38	54, 45	16.16, 12.46	12.13, 11.46	0.42	0.50	0.05	0.29	0.316	1.34
	Covert aggression	ag.c_SHRP	100, 38	54, 45	16.16, 12.46	12.13, 11.46	0.45	0.56	0.07	0.15	0.692	1.07
	Hostility	hos_SHRP	100, 38	54, 45	16.16, 12.46	12.13, 11.46	0.44	0.60	0.14	0.20	0.617	1.95
	Physical aggression	ag.p_SHRP	100, 38	54, 45	16.16, 12.46	12.13, 11.46	0.31	0.38	0.04	0.67	**0.021***	0.74
**Affective Reactivity Index (ARI)**	Total	tot_ARI	101, 38	59, 46	16.13, 12.46	13.14, 12.37	0.40	0.49	0.06	0.17	0.511	1.54
**Conners-3 (CON)**	Inattention	att_CON	63, 38	48, 44	13.15, 12.46	11.19, 12.2	0.43	0.55	0.09	0.20	0.502	1.05
	Hyperactivity/Impulsivity	hyp.imp_CON	63, 38	48, 44	13.15, 12.46	11.19, 12.2	0.47	0.46	-0.04	0.09	0.540	1.34
	Learning problems	learn_CON	63, 38	48, 44	13.15, 12.46	11.19, 12.2	0.24	0.26	0.00	0.45	0.478	0.30
	Executive functioning	exec_CON	63, 38	48, 44	13.15, 12.46	11.19, 12.2	0.33	0.37	0.02	0.21	0.699	0.11
	Defiance	def_CON	63, 38	48, 44	13.15, 12.46	11.19, 12.2	0.54	0.51	-0.06	0.12	0.418	1.76
	Peer relations	peer_CON	63, 38	48, 44	13.15, 12.46	11.19, 12.2	0.50	0.50	-0.04	0.31	0.259	2.79
	Total	tot_CON	63, 38	48, 44	13.15, 12.46	11.19, 12.2	0.56	0.62	0.02	0.13	0.804	1.09
	ADHD inattentive subtype	adhd.att_CON	63, 38	48, 44	13.15, 12.46	11.19, 12.2	0.43	0.52	0.06	0.10	0.991	0.80
	ADHD hyperactive subtype	adhd.hyp_CON	63, 38	48, 44	13.15, 12.46	11.19, 12.2	0.44	0.42	-0.05	0.10	0.498	1.07
	Conduct disorder	cond_CON	63, 38	48, 44	13.15, 12.46	11.19, 12.2	0.50	0.43	-0.11	0.25	**0.047***	1.20
	Oppositional defiant disorder	odd_CON	63, 38	48, 44	13.15, 12.46	11.19, 12.2	0.44	0.56	0.09	0.07	0.977	1.47
**Developmental Coordination Disorders Questionnaire (DCD; inverted)**	Control	cont_DCD	100, 38	62, 51	16.16, 12.46	12.77, 11.7	0.37	0.32	-0.08	0.18	0.337	1.66
	Fine Motor	fine_DCD	100, 38	62, 51	16.16, 12.46	12.77, 11.7	0.29	0.34	0.04	0.35	0.134	0.91
	Coordination	coord_DCD	100, 38	62, 51	16.16, 12.46	12.77, 11.7	0.37	0.47	0.07	0.25	0.087	0.72
	Total	tot_DCD	100, 38	62, 51	16.16, 12.46	12.77, 11.7	0.41	0.49	0.05	0.17	0.069	1.41
**Social Communication Questionnaire (SCQ)**	Total	tot_SCQ	100, 37	62, 50	16.16, 12.38	12.77, 11.62	0.37	0.38	-0.02	0.09	0.719	3.04
	Reciprocal social interaction	soc_SCQ	100, 37	62, 50	16.16, 12.38	12.77, 11.62	0.33	0.31	-0.04	0.09	0.864	3.91
	Communication	com_SCQ	100, 37	62, 50	16.16, 12.38	12.77, 11.62	0.26	0.33	0.05	0.11	0.873	1.18
	Repetitive Behavior	rep_SCQ	100, 37	62, 50	16.16, 12.38	12.77, 11.62	0.29	0.25	-0.07	0.29	0.206	1.75
**Strength and Difficulties Questionnaire (SDQ)**	Emotional symptoms	emo_SDQ	101, 37	50, 51	16.13, 12.38	10.72, 11.7	0.34	0.33	-0.03	0.19	0.897	0.67
	Conduct problems	cond_SDQ	101, 37	50, 51	16.13, 12.38	10.72, 11.7	0.41	0.51	0.07	0.15	0.133	0.35
	Hyperactivity/Innatention	hyp.imp_SDQ	101, 37	50, 51	16.13, 12.38	10.72, 11.7	0.42	0.49	0.04	0.18	0.281	1.15
	Peer relationship problems	peer_SDQ	101, 37	50, 51	16.13, 12.38	10.72, 11.7	0.36	0.43	0.04	0.27	0.334	2.14
	Prosocial behavior (inverted)	soc_SDQ	101, 37	50, 51	16.13, 12.38	10.72, 11.7	0.34	0.38	0.02	0.69	0.081	0.83
	Total difficulties	tot_SDQ	101, 37	50, 51	16.13, 12.38	10.72, 11.7	0.57	0.67	0.07	0.26	0.267	1.25
**Social Responsiveness Scale (SRS)**	Total score	tot_SRS	101, 38	62, 51	16.13, 12.46	12.77, 11.7	0.65	0.64	-0.05	0.09	0.133	1.93
	Social awareness	awr_SRS	101, 38	62, 51	16.13, 12.46	12.77, 11.7	0.39	0.49	0.08	0.14	0.751	0.98
	Social cognition	cog_SRS	101, 38	62, 51	16.13, 12.46	12.77, 11.7	0.55	0.56	-0.02	0.13	0.810	0.75
	Social communication	com_SRS	101, 38	62, 51	16.13, 12.46	12.77, 11.7	0.61	0.61	-0.03	0.12	0.143	2.09
	Social motivation	mot_SRS	101, 38	62, 51	16.13, 12.46	12.77, 11.7	0.39	0.31	-0.11	0.28	**0.026***	0.76
	Restricted interests/repetitive behavior	man_SRS	101, 38	62, 51	16.13, 12.46	12.77, 11.7	0.55	0.51	-0.08	0.18	0.442	3.73
	ASD social communication/interaction	asd_SRS	101, 38	62, 51	16.13, 12.46	12.77, 11.7	0.63	0.64	-0.03	0.09	0.132	1.59
**Child Behavior Checklist (CBCL)**	Anxious/Depressed	ax.dep_CBCL	61, 37	47, 46	12.88, 12.6	11.04, 12.37	0.45	0.30	-0.17	0.25	0.578	0.27
	Withdrawn/Depressed	wt.dep_CBCL	61, 37	47, 46	12.88, 12.6	11.04, 12.37	0.43	0.27	-0.19	0.37	0.231	1.96
	Somatic complaints	som_CBCL	61, 37	47, 46	12.88, 12.6	11.04, 12.37	0.31	0.28	-0.06	0.49	0.097	1.34
	Social problems	soc_CBCL	61, 37	47, 46	12.88, 12.6	11.04, 12.37	0.59	0.58	-0.05	0.54	**0.009***	4.06
	Thought problems	tht_CBCL	61, 37	47, 46	12.88, 12.6	11.04, 12.37	0.54	0.42	-0.16	0.51	0.286	1.58
	Attention problems	att_CBCL	61, 37	47, 46	12.88, 12.6	11.04, 12.37	0.47	0.55	0.05	0.20	0.672	1.44
	Rule breaking	rule_CBCL	61, 37	47, 46	12.88, 12.6	11.04, 12.37	0.39	0.48	0.07	0.12	0.700	0.12
	Aggressive behavior	ag_CBCL	61, 37	47, 46	12.88, 12.6	11.04, 12.37	0.58	0.59	-0.02	0.13	0.129	0.99
	Internalizing problems	int_CBCL	61, 37	47, 46	12.88, 12.6	11.04, 12.37	0.51	0.39	-0.15	0.28	0.279	1.64
	Externalizing problems	ext_CBCL	61, 37	47, 46	12.88, 12.6	11.04, 12.37	0.59	0.61	-0.01	0.09	0.463	0.92
	Total problems	tot_CBCL	61, 37	47, 46	12.88, 12.6	11.04, 12.37	0.70	0.70	-0.04	0.36	0.062	2.00

Table S1: Supplemental information from all analyses. Including: instrument abbreviation, subscales, and variable names; sample size per instrument in each group (XXY/KS, XYY, and matched XY groups); mean age of respondents per instrument and group; global coupling (scale degree) scores per SCA group and their Deming residual; divergence score per scale and its *p*-value resulting from permutation against the null distribution; and the absolute difference in effect size between groups (XXY/KS - XYY). Bolding and asterisk (*) denotes nominal statistical significance ($p < .05$). Bolding and double asterisk (**) denotes statistical significance after Bonferroni correction for multiple comparisons ($p < .05/53$, or $p < .0009$).
